# Circ_0001715 accelerated lung adenocarcinoma process by the miR-1322/CANT1 axis

**DOI:** 10.1186/s13000-023-01348-2

**Published:** 2023-08-08

**Authors:** Yue Niu, Lina Fan, Xiaoyu Shi, Jia Wu, Tengqi Wang, Xiaofeng Hou

**Affiliations:** 1 Department of Oncology, Bayannur Hospital, No.98 Ulanbuhe Road, Linhe District, Bayannaoer City, Inner Mongolia Province 015000 PR China; 2 Department of Gastrointestinal Surgery, Bayannur Hospital, No.98 Ulanbuhe Road, Linhe District, Bayannaoer City, Inner Mongolia Province 015000 PR China

**Keywords:** LUAD, circ_0001715, miR-1322, CANT1

## Abstract

Lung adenocarcinoma (LUAD) is a type of lung cancer, which belongs to non-small cell lung cancer and has seriously endangered the physical and mental health of people. The study of circRNAs (circRNAs) has been increasingly hot in recent years, in which circRNAs also play an important regulatory role in cancer. The aim of this study was to investigate the biological molecular mechanisms of circ_0001715 in the progression of LUAD. The expression of circ_0001715, miR-1322 and calcium-activated nucleotidase 1 (CANT1) in LUAD tissues and cell lines was assessed by quantitative reverse transcription PCR (RT-qPCR) and western bot assay. Clone formation assay, 5-Ethynyl-2’-Deoxyuridine (EDU) assay and wound healing assay were used to verify the proliferation ability of cells. Dual-luciferase reporter assay and RNA pull-down assay were performed to characterize the interactions between the three factors. Finally, a mouse tumor model was constructed to assess the tumorigenicity of circ_0001715. RT-qPCR assay results showed that circ_0001715 expression was significantly increased in LUAD tissues and cell lines. Finally, knockdown of circ_0001715 could inhibit tumor growth in vivo. Circ_0001715 regulated the progression of LUAD through the miR-1322/CANT1 axis. The results of this study provided ideas for understanding the molecular mechanisms of circ_0001715 in LUAD.

**Supplementary Information:**

The online version contains supplementary material available at 10.1186/s13000-023-01348-2.

## Introduction

Lung adenocarcinoma (LUAD), a type of non-small cell lung cancer, is one of the biggest problems that affect the physical and mental health of people all over the world and is the most important cause of death from cancer worldwide [1–3]. LUAD originates from bronchial epithelial cells [4] and is predominantly found in female patients and non-smoking patients [5]. In the early stages of cancer, although the treatment is more effective and the prognosis is more obvious, it is difficult to monitor the progression of the disease in the early stages and it is also easy to recur [6]. In the advanced stage, cancer has reached a strongly aggressive process [7], which is difficult to treat and has a very poor prognosis with a very high mortality rate [6, 8]. However, we do not know much about the biomolecular mechanisms involved in the development of LUAD. Our study aims to investigate the pathogenesis in the progression of LUAD, in order to investigate the biomarkers that can detect LUAD earlier, and provide strategies to identify more effective therapeutic measures.

Circular RNAs (circRNAs) are a special class of closed RNA molecules [9] that are more stable and less prone to degradation [10]. Emerging advances have demonstrated their activity as microRNA (miRNA) sponges and protein decoys, and some circRNAs even act as translation templates in multiple pathophysiological processes [11]. In recent years, many studies have reported that circRNAs were dysregulated in disease and play an important regulatory role in disease progression [12]. To name a few: in the development of gastric cancer, the expression of circ_0061137 was aberrant and significantly reduced in GC tissues and cells, and overexpression of circ_0061137 could inhibit cell proliferation, migration and invasion while also inducing apoptosis, therefore, the inhibitory role of circ_0061137 in tumor development could be used as a potential biomarker for GC [13], circ_0071127 was reduced in expression in breast cancer and could further regulate HRD1 expression by sponging miR-513a-3p [14]. In addition to that, Guo et al. found that circ_0001715 was significantly highly expressed in LUAD tissues [15]. However, the regulatory mechanism of circ_0001715 in the development of lung adenocarcinoma in the molecular mechanism was not clear, so we carried out a functional study of circ_0001715 in LUAD, as well as its downstream regulatory mechanism.

MicroRNAs (miRNAs) are a class of non-coding single-stranded RNA molecules that are produced by encoding endogenous genes [16]. MiRNAs also have an important regulatory role in the development of cancer. And circRNAs can act as sponges of miRNAs to further regulate cancer development [17, 18]. For instance, in pancreatic cancer progression, circ_0086375 could function as a sponge for miR-486-5p and could regulate the process of pancreatic cancer progression through miR-486-5p [18], circ-AKT3 could act as a sponge for miR-296-3p to further regulate the development of renal cell carcinoma [17]. It has been shown that miR-1322 was significantly under-expressed in the development of LUAD [19], and we aimed to investigate the interrelationship between circ_0001715 and miR-1322.

Calcium-activated nucleotidase 1 (CANT1), belonging to the apyrase family, is a calcium-dependent enzyme [20]. It has high sequence homology with decarboxylase genes and exhibits a preference for uridine diphosphate (UDP) [21]. CANT1 has been revealed to exert a crucial role in endochondral ossification and cartilage proteoglycan synthesis [22]. Moreover, the relationship between CANT1 and tumor growth has been reported in several cancers. For example, CANT1 might be a prognostic biomarker in the primary prostate [23]. Also, CANT1 downregulation decreased cell invasion and migration in clear cell renal cell cancer [24]. In lung cancer, CANT1 facilitated cell malignant behaviors by activating the NF-ĸB signaling [25]. Bioinformatics predictions show a targeted relationship between CANT1 and miR-1322, but whether they have a relationship in LUAD needs to be verified.

In this study, we detected significant high expression of circ_0001715 in LUAD tissues as well as in cell lines by RT-qPCR. Further detection of the function of circ_0001715 in LUAD cells showed that circ_0001715 promoted cell progression. During the mechanism exploration, we found that circ_0001715 could act as a sponge for miR-1322 to further upregulate CANT1 expression, thus promoting the proliferative process of LUAD. We conjecture that circ_0001715 could serve as an effective biomarker to provide an efficient strategy for disease diagnosis and treatment.

## Materials and methods

### Tissues collection

The tissues in this exploration included 43 pairs of tumor tissues, as well as their paired normal tissues that were derived from LUAD patients. These patients were all from Bayannur Hospital. All tissue samples needed to be stored in liquid nitrogen immediately after removal and finally stored in a -80 °C refrigerator (AIPUINS, Hangzhou, China). All patients were not treated with radiotherapy and chemotherapy prior to surgery and signed an informed consent. The clinicopathological parameters of LUAD patients are displayed in Table 1. The study met the criteria of Xiantao first people’s Hospital of Yangtze University and was approved by Bayannur Hospital Ethics Committee.

**Table 1:** Correlation between circ_0001715 expression and clinicopathological parameters of lung adenocarcinoma patients

Clinical feature		circ_0001715		
	n	High	Low	*P* -Value
Age		22	21	0.666
≥55	28	15	13	
<55	15	7	8	
Gender				0.451
Man	23	13	10	
Woman	20	9	11	
TNM state				0.044*
III	27	17	10	
I-II	16	5	11	
Lymph node metastasis				0.009*
Negative	18	5	13	
Positive	25	17	8	

*P < 0.05, statistically significant

### Cells culture

Human normal lung epithelial cells: BEAS-2B (MXC048, Mxbio, Shanghai, China) and LUAD cancer cell lines: A549 (CL-0016, Procell, Wuhan, China) and H1299 (Afzhan, Shanghai, China). BEAS-2B was cultured with Roswell Park Memorial Institute (RPMI-1640, Gibco, Carlsbad, CA, USA) + 10% fetal bovine serum (FBS, Gibco). A549 and H1299 were cultured with dulbecco’s modified eagle medium (DMEM; Gibco) + 10% FBS. All cells were cultured at 37 °C in a 5% CO_2_ incubator.

### Reverse transcription-quantitative PCR (RT-qPCR)

For total RNA extraction, RNA was isolated from cells using Trizol reagent (Invitrogen, Carlsbad, CA, USA). After extraction of total RNA, the extracted total RNA was reverse transcribed into cDNA using a reverse transcription kit (Invitrogen). Fluorescent quantitative PCR was performed using the SYBR Premix EX Taq kit (Takara, Japan). β-actin was used as an internal reference for genes and U6 was used as an internal reference for miRNAs. Finally, all genes expression were quantified using the − 2 ^ΔΔCt^ method. The specific primers were listed in Table 2.

**Table 2:** Primers sequences used for PCR.

Name		Primers for PCR (5’-3’)
hsa_circ_0001715	Forward	GTTCATCGGGGTGCTCTACAA
	Reverse	GCAGCTCCTCCATGCTCTTGAT
CANT1	Forward	AGTCGGCCACCTTCCTCC
	Reverse	AACGTGGGAAGCTGAGTGAG
miR-1322	Forward	GCCGAGGATGATGCTGCTGATG
	Reverse	CTCAACTGGTGTCGTGGAG
β-actin	Forward	CTTCGCGGGCGACGAT
	Reverse	CCACATAGGAATCCTTCTGACC
U6	Forward	CTCGCTTCGGCAGCACA
	Reverse	AACGCTTCACGAATTTGCGT

### Clone formation assay

Cells transfected with plasmids were inoculated in 12-well plates with 5 × 10^4^ cells per well. After inoculation, the 12-well plates were placed in an incubator, and the medium was changed every two days. When colonies were formed, the medium was discarded and methanol fixative (Beyotime, Shanghai, China) was added into the well, followed by colony staining using crystal violet (Beyotime). Finally, the colonies were counted and recorded.

### 5-Ethynyl-2ʹ-Deoxyuridine (EDU) assay

A549 and H1299 cells were inoculated in 24-well plates (5 × 10^4^ cells/well). Cells were labeled by co-incubation with EDU reagent (C10310, Biomart, Guangzhou, China) for 2 h. After incubation, the cells were washed with medium and the nuclei were stained with DAPI for 30 min. After staining, the labeled cells were fixed with 4% paraformaldehyde. Finally, the cells were photographed under an inverted microscope.

**Fig. 1:**
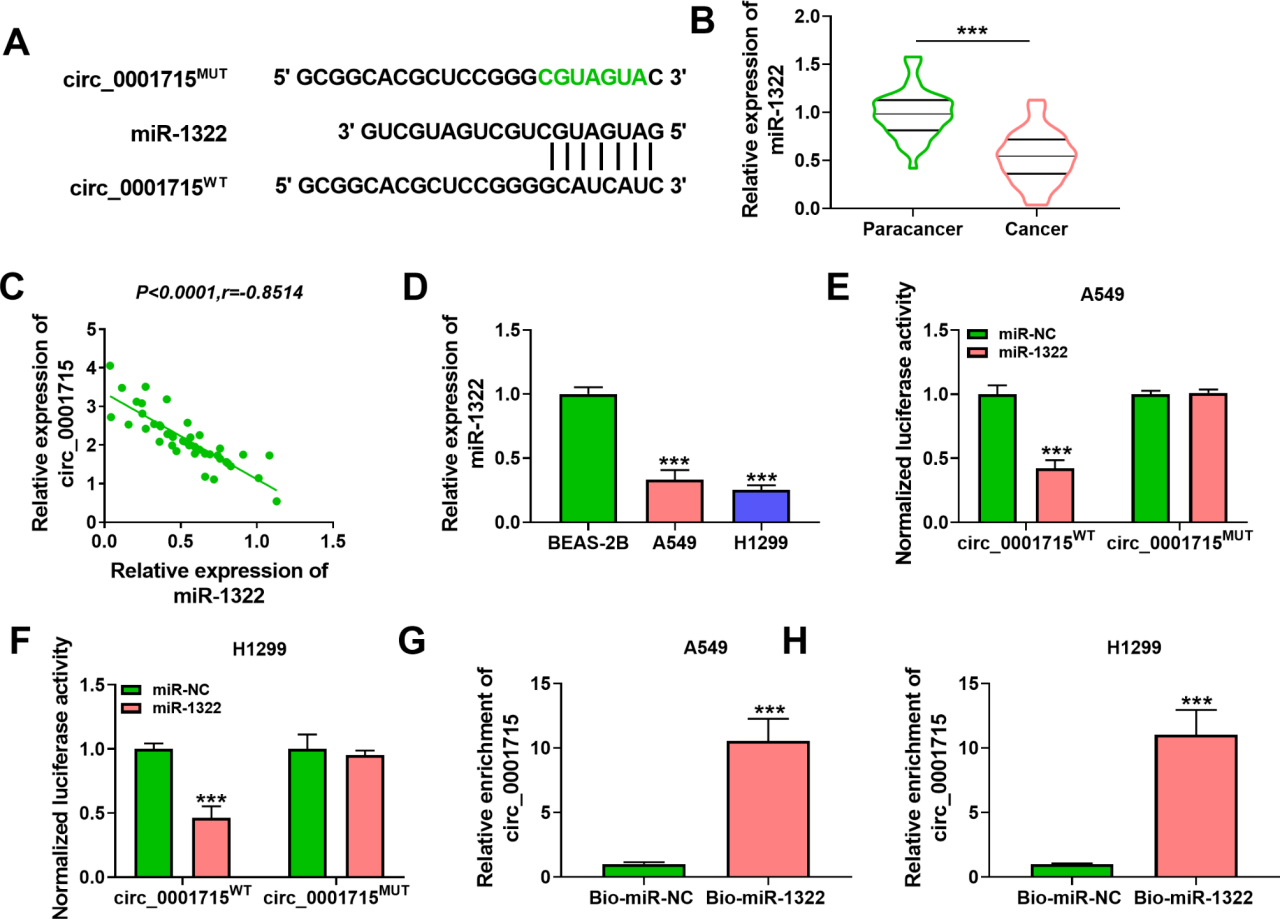
**Circ_0001715 expression was increased in LUAD tissues and cells.** The chromosome position and sequence of circ_0001715 (A). Circ_0001715 expression was detected in LUAD tissues and cell lines by RT-qPCR (B and C). ***p < 0.001

### Wound healing assay

The corresponding plasmids were transfected into cells and then inoculated in 6-well plates with 10^5^ cells per well. After the cells adhered to the 6-well plates, a scratch was made horizontally using the tip of the pipette tip at 0 h. The cells at 0 h were photographed and processed using an inverted microscope. After 24 h of cell culture, the cells were further photographed and stored using an inverted microscope for 24 h. The distance of induced damage of cells at both time periods was measured, and the cells at 0 h were treated as a control for normalization.

**Fig. 2:**
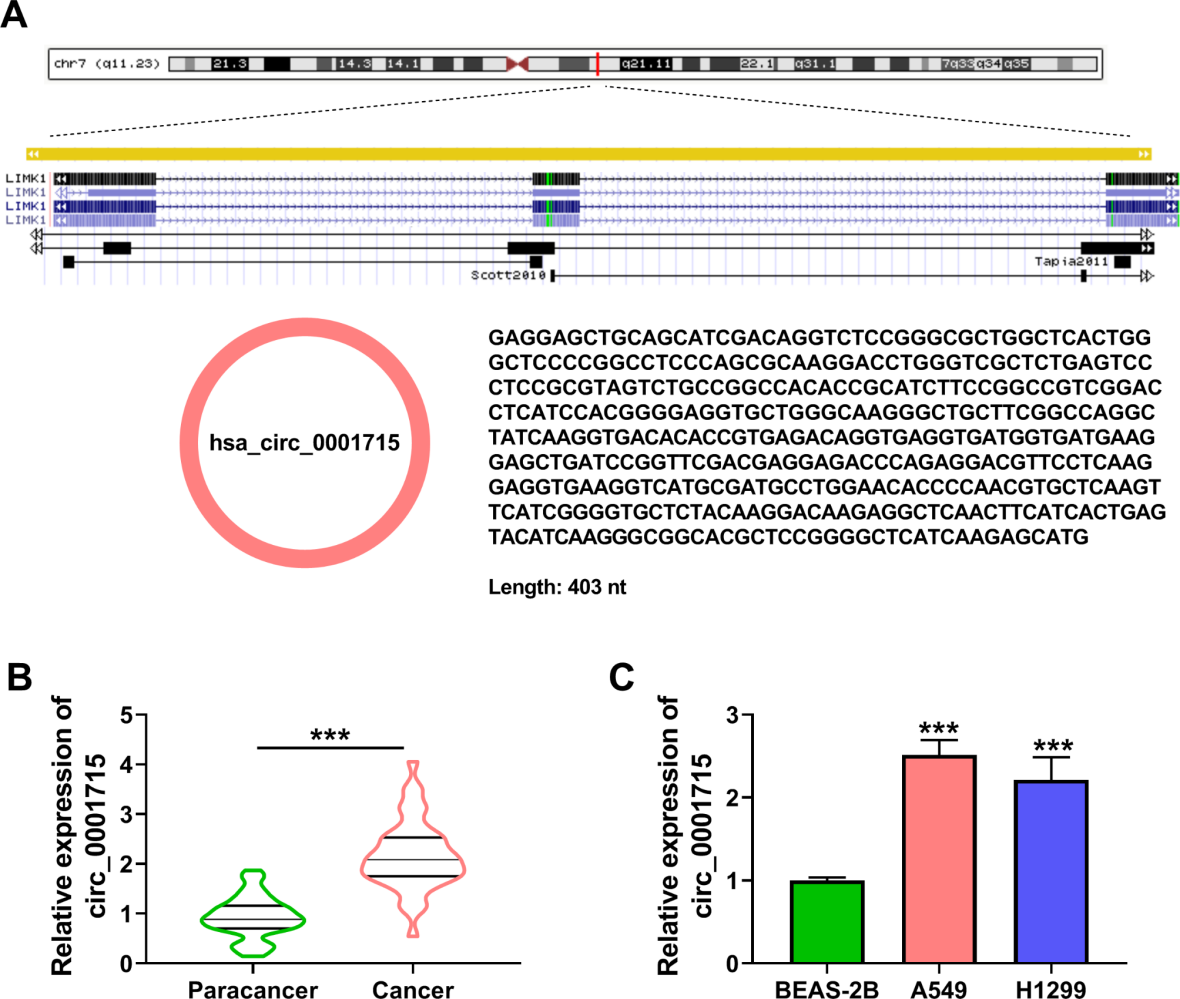
**Knockdown of circ_0001715 inhibited the biological properties of A549 and H1299 cells.** The expression of circ_0001715 was assessed by RT-qPCR (A). Clone formation assay was performed to investigate the effects of circ_0001715 knockdown on the proliferation abilities of A549 and H1299 cells (B). The effect of circ_0001715 knockdown on cell proliferation was detected by EDU assay and wound healing assay (C and D). The sphere formation efficiency of A549 and H1299 cells was detected by sphere formation assay (E). The apoptotic rates were performed and analyzed after transfection of si-circ_0001715 in A549 and H1299 cells (F). The protein levels of PCNA and Bax were detected by western blot assay in each group (G and H). The tube formation ability of HUVECs was determined by tube formation assay (I). ***p < 0.001

### Sphere formation assay

A549 and H1299 cells were inoculated in a 24-well ultra-low adsorption microplate (Corning, NY, USA) (5 × 10^4^ cells/well) for culture. Cells were cultured in serum-free DMEM with the addition of epidermal growth factor (EGF, ACROBiosystems, Beijing, China), basic fibroblast growth factor (bFGF, ACROBiosystems) and penicillin/streptomycin (beyotime). After one week of culture, the formed spheres were imaged and photographed using an inverted microscope, and then the images were measured and calculated.

**Fig. 3:**
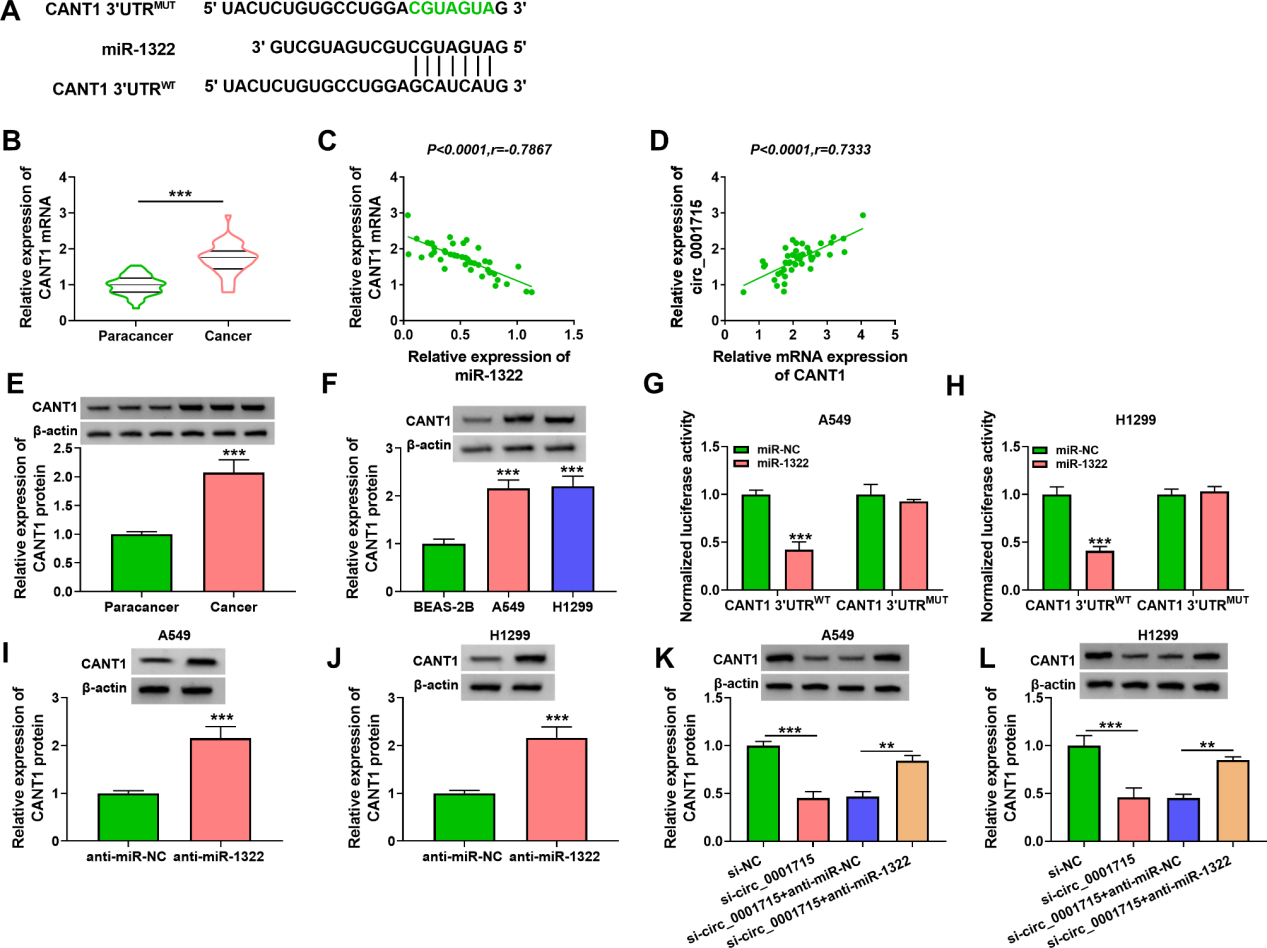
**Circ_0001715 sponged miR-1322 in A549 and H1299 cells.** The predicted binding site between miR-1322 and circ_0001715 was shown. The mutated (MUT) version of circ_0001715 is presented (A). The expression of miR-1322 was detected in LUAD tissues by RT-qPCR (B). The correlation between miR-1322 and circ_0001715 in LUAD tissues was determined by Pearson’s correlation analysis (C). Expression of miR-1322 was detected in LUAD cell lines by RT-qPCR (D). Relative luciferase activity was assessed in A549 and H1299 cells transfected with miR-1322 or miR-NC and circ_0001715 WT or circ_0001715 MUT (E and F). The relative enrichment of circ_0001715 was detected by RNA pull-down assay (G and H). ***p < 0.001

### Flow cytometry assay

Cells were inoculated in 6-well plates (8 × 10^5^ cells/well). After cell treatment, all cells were collected by washing with cold PBS and then digestion with trypsin (Gibco). Further, 5 µL Annexin V-FITC and 5 µL PI (BB-4101, BestBio, Shanghai, China) were added to the resuspended cytosol for co-incubation with cells. The incubation was carried out at room temperature and protected from light for 30 min, and the cells were detected by a flow cytometer (Becton, Franklin Lakes, NJ, USA).

**Fig. 4:**
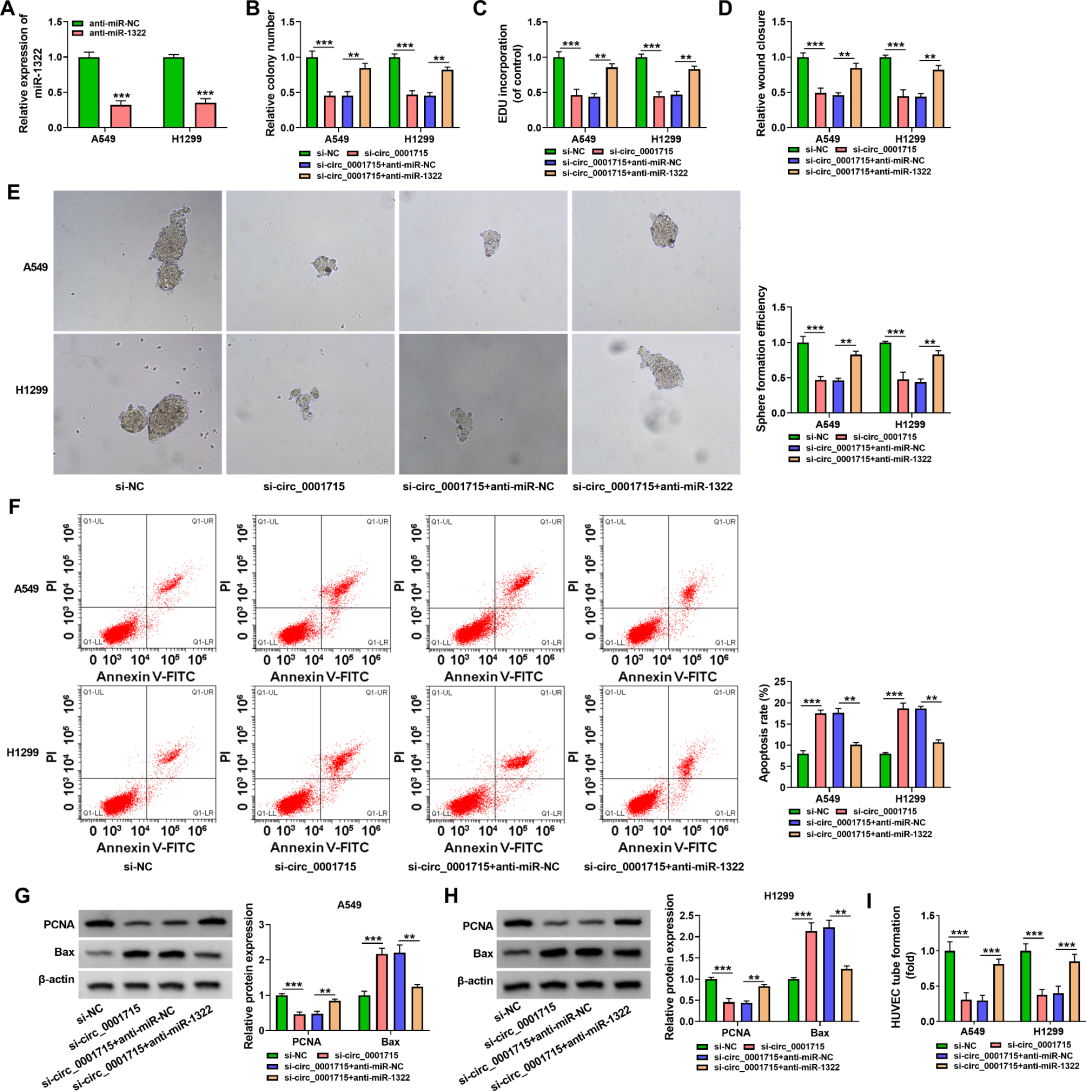
**Inhibition of miR-1322 reversed the effects of circ_0001715 knockdown on NSCLC cell proliferation and apoptosis in A549 and H1299 cells.** The expression of miR-1322 was assessed by RT-qPCR after transfection of anti-miR-1322 and anti-miR-NC (A). The proliferation ability of A549 and H1299 cells that transfected with si-NC, si-circ_0001715, si-circ_0001715 + anti-NC, si-circ_0001715 + anti-miR-1322 was detected by the clone formation assay, EDU assay and wound healing assay (B-D). Sphere formation assay was applied for rescue experiments in A549 and H1299 cells to assess whether the biological function of circ_0001715 in cells could be affected by miR-1322 after transfected with si-NC, si-circ_0001715, si-circ_0001715 + anti-NC, si-circ_0001715 + anti-miR-1322 (E). Apoptosis rate of A549 and H1299 cells were presented by flow cytometry assay transfected with si-NC, si-circ_0001715, si-circ_0001715 + anti-NC, si-circ_0001715 + anti-miR-1322 (F). Western blotting assay showed that circ_0001715 silencing on PCNA and Bax in A549 and H1299 cells was reversed by miR-1322 inhibitor transfected with si-NC, si-circ_0001715, si-circ_0001715 + anti-NC, si-circ_0001715 + anti-miR-1322 (G and H). Effect of the above cell lines on the tube formation ability of HUVECs was determined by tube formation assay (I). **p < 0.01, ***p < 0.001

### Western bot assay

All cells were enriched by cell scraping, and RIPA Lysis Buffer (Boster, Wuhan, China) was added to the enriched cells for isolating total proteins. After low-temperature centrifugation, the supernatant was collected and the protein concentration was determined by the BCA method. Then, 50 µg protein was separated on SDS-PAGE. After electrophoretic, the protein bands were separated onto the PVDF membrane. Skim milk was used to seal the membrane at room temperature for 1.5 h. Primary antibodies were used to incubate with PVDF membrane overnight at 4 °C. Primary antibody: proliferating cell nuclear antigen (PCNA, ab29, Abcam, Cambridge, MA, USA), BCL2 associated X, apoptosis regulator, (Bax, ab182733, Abcam), calcium-activated nucleotidase 1 (CANT1, ICA357Mu01, LMAI Bio, Shanghai, China) and β-actin (ab8226, Abcam). After the primary antibody was incubated, the PVDF membrane was cleaned 3 times with TBST, and then co-incubated with the secondary antibody (goat anti-mouse (ab216772)/rabbit (ab97051) IgG H&L) for 1 h at room temperature. Finally, the ECL kit (1FF7872, Bioon, Shanghai, China) was used for developing the blots.

**Fig. 5:**
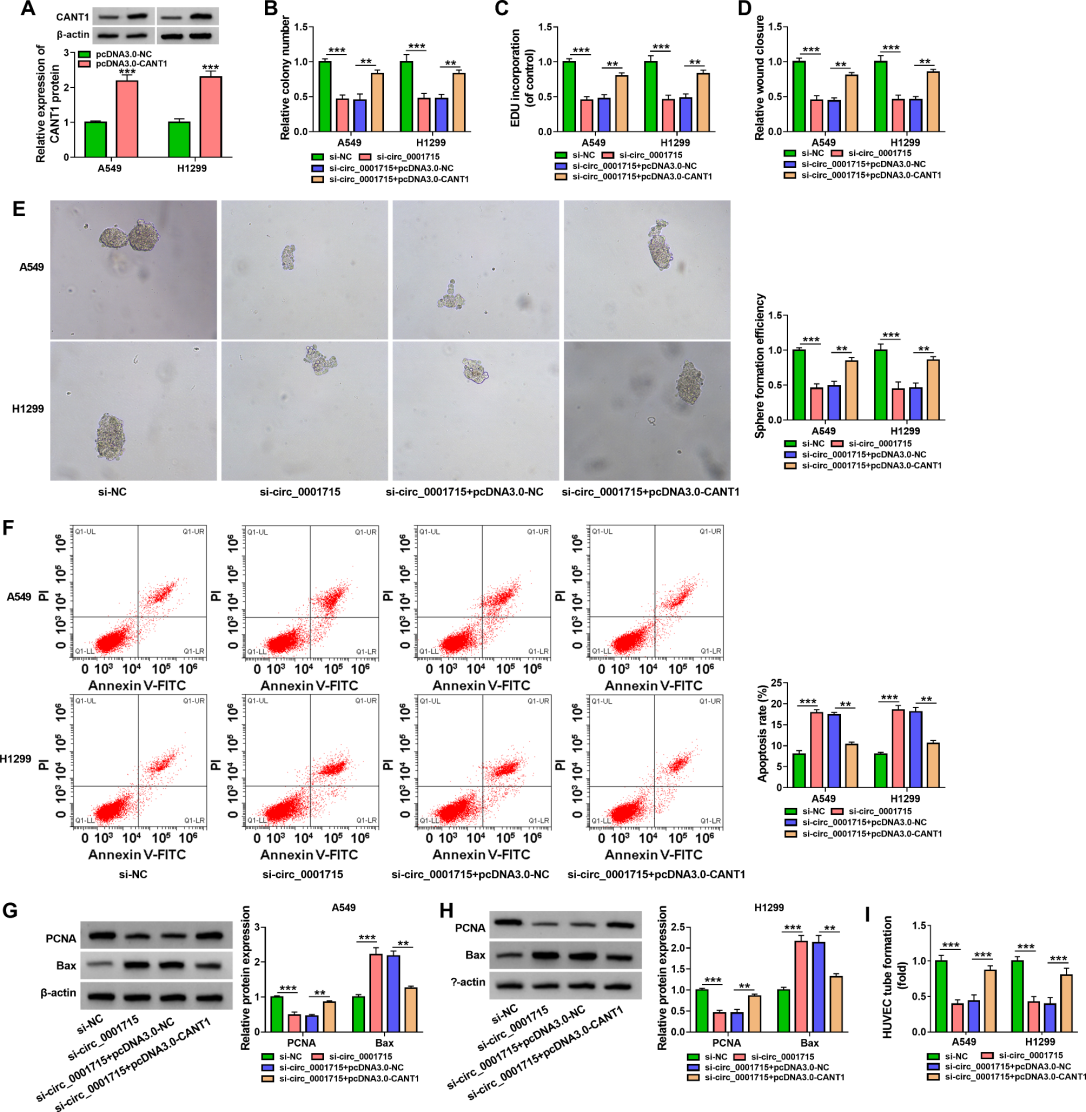
**CANT1 was a target gene of miR-1322 and circ_0001715 could regulate the expression of CANT1 through miR-1322.** The putative binding sites of miR-1322 on CANT1 were shown (A). The expression of CANT1 was measured by RT-qPCR and western blotting assay (B). The correlation between CANT1 and miR-1322 or circ_0001715 in LUAD tissues was determined by Pearson’s correlation analysis (C and D). CANT1 protein levels in LUAD tissues and cell lines were detected by RT-qPCR (E and F). Dual-luciferase reporter assay was used to demonstrate the relationship between miR-1322 and CANT1 (G and H). The expression of CANT1 was assessed by western blotting assay after transfection of anti-miR-1322 (I and J). The effect of circ_0001715 silencing and miR-1322 inhibitor on the protein level of CANT1 in A549 and H1299 cells was illustrated by western blot assay (K and L). **p < 0.01, ***p < 0.001

### Tube formation assay

24-well plates coated with 250 µL of Matrigel (BD Bioscience, USA) were placed in an incubator for half an hour. Approximately 1 × 10^5^ HUVECs were seeded into each well supplemented with 200 µL of the conditioned medium in which LUAD cell lines were incubated. After 8 h of incubation, capillary structures were photographed under a ×100 microscope.

### Dual-luciferase reporter assay

Circ_0110757^WT^, circ_0110757^MUT^, CANT1-3’UTR^WT^ and CANT1-3’UTR^MUT^ (WT: wild type; MUT: mutant) were cloned into the pmirGLO plasmid to construct the corresponding dual luciferase reporter plasmids. The constructed plasmids were co-transfected with miR-1322 mimics into A549 and H1299 cells for 48 h. The luciferase activity was further examined using the Dual-Luciferase Reporter System (Promega, Carlsbad, CA, USA).

### RNA pull-down assay

Streptavidin magnetic beads were incubated with a biotin-labeled miR-1322 probe or circ_0001715 probe. Then, streptavidin magnetic beads-RNA complexes were co-incubated with cells (ThermoFisher, Shanghai, China) for 3 h at room temperature, followed by incubating with RIP elution buffer to elute the protein-RNA complex from the surface of the beads. Finally, RNA was detected by using RT-qPCR.

### Immunohistochemistry (IHC) assay

The tumor tissue samples were taken out, cleaned with PBS for 3 times, and then dried with filter paper for section processing. After sectioning, dewaxing and antigen repair were performed. Next, the sections were sealed with FBS. After the sealing, the sections were incubated with primary antibody Ki-67 (1:200, Abcam) for overnight incubation at 4℃. The second antibody was added and incubated at room temperature for 30 min. After that, the sections were cleaned with PBS for 3 times, and then a chromogenic agent was added. Hematoxylin was then used to stain the sections, and the sections were sealed. After drying, the images were collected by microscope for analysis.

**Fig. 6:**
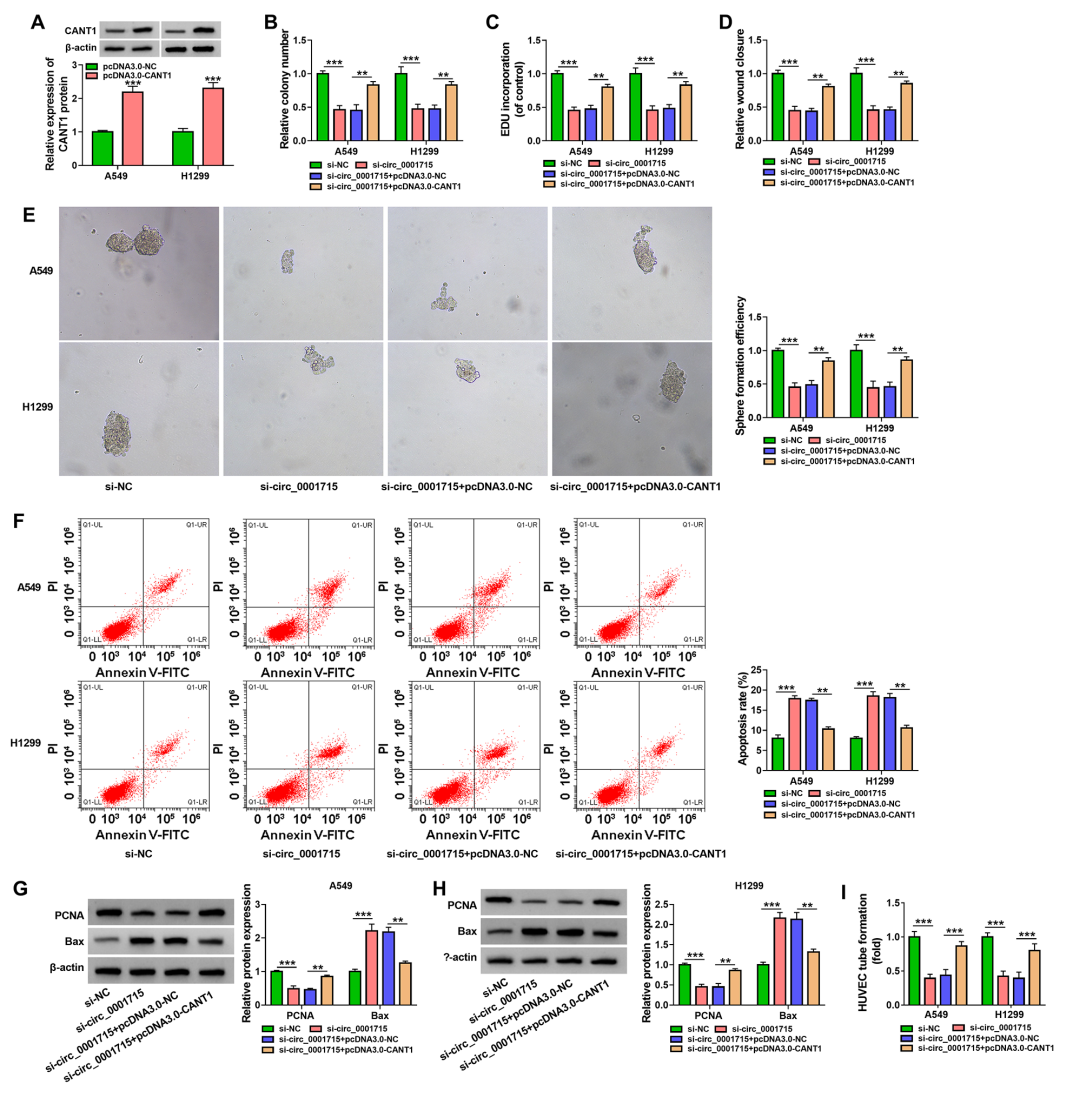
**Overexpression of CANT1 rescued the effect of circ_0001715 silencing on A549 and H1299 cell progression.** The overexpression efficiency of CANT1 was determined by western blot assay in A549 and H1299 cells (A). Clone formation assay and EDU assay were employed to illustrate the effects between circ_0001715 knockdown and CANT1 overexpression on cell proliferation in cells (B and C). The proliferation capacity of the cells was further verified using wound healing assay (D). Sphere formation efficiency is tested by sphere formation assay after transfection with si-circ_0001715 and pcDNA3.0-CANT1 in cell lines (E). Flow cytometry assay was used to determine the function between si-circ_0001715 and pcDNA3.0-CANT1 on the apoptosis of A549 and H1299 cells (F). The impacts were measured between circ_0001715 knockdown and CANT1 overexpression on PCNA and Bax protein expression by western blot assay (G and H). Effect of the above cell lines on the tube formation ability of HUVECs was determined by tube formation assay (I). Figure B-I in all experimental cells transfected with si-NC, si-circ_0001715, si-circ_0001715 + pcDNA3.0-NC, si-circ_0001715 + pcDNA3.0- CANT1. **p < 0.01, ***p < 0.001

### Xenografts assay

The A549 cells of stably knocked down circ_0001715 (sh-circ_0001715) and control (sh-NC) were inoculated subcutaneously into four-week-old BALB/c nude mice (5/group). Tumor volume (length × width 2 × 0.5) was monitored once a week and recorded. At the fifth week after volume measurement, the mice were sacrificed, the tumor was removed and body weight was measured and recorded. All nude mice were purchased from Vital River Laboratory Animal Technology (Beijing, China). All animal experiments were approved by Bayannur Hospital Research Animal Care and Use Committee.

### Statistical analysis

All experimental results in this study were summarized and statistically analyzed using GraphPad Prism 8 software (SanDiego, CA, USA). Each experiment was repeated three times. During the analysis, the student *t*-test was used to evaluate the significance analysis between the two groups; significance analysis for more than two groups was assessed using one-way ANOVA or two-way ANOVA. The *p-*value was statistically significant only when it was less than 0.05.

## Results

### Circ_0001715 was increased in LUAD tissues and cell lines

In order to explore the role of circ_0001715 in the development of LUAD, we detected the expression level of circ_0001715 in LUAD tissues and cell lines. Firstly, the chromosome location and sequence details of circ_0001715 were shown in Fig. 1A. As shown in Fig. 1B, the expression of circ_0001715 in LUAD tissues was significantly higher than that in adjacent normal tissues. High circ_0001715 expression was associated with TNM stage and lymph node metastasis of LUAD patients (Table 1). Similarly, circ_0001715 was also significantly up-regulated in A549 and H1299 cells compared with normal control cells (Fig. 1C). The circular characteristics of circ_0001715 in LUAD cell lines were validated by RNase R digestion, and the results indicated that circ_0001715 was more stable than linear LIMK1 mRNA (Supplementary Fig. 1A and B). These results suggested that the abnormal expression of circ_0001715 might be involved in the progression of LUAD.

### Circ_0001715 promoted proliferation and inhibited apoptosis of LUAD cell lines

We further explored the effect of circ_0001715 on the progression of LUAD cells. Firstly, si-circ_0001715 was designed and transfected into the cells to examine the knockdown efficiency, and the results showed that the expression of circ_0001715 was significantly downregulated in the circ_0001715 knockdown group in A549 and H1299 cells (Fig. 2A). Next, the silencing of circ_0001715 significantly inhibited the colony formation ability, EDU incorporation rate and cell migration ability, these results indicated that knockdown of circ_0001715 significantly inhibited A549 and H1299 cell proliferation (Fig. 2B and D). The sphere formation assay results showed that sphere formation efficiency in A549 and H1299 cells was significantly reduced in the circ_0001715 silence group (Fig. 2E). Flow cytometry assay also explained that downregulation of circ_0001715 could induce the apoptosis of A549 and H1299 cells (Fig. 2F). Western bot assay analysis showed that the reduction of circ_0001715 expression inhibited the expression of PCNA in LUAD cell lines, while promoting the expression of Bax (Fig. 2G H). Tube formation assay showed that LUAD cell lines with circ_0001715 silencing reduced the tube formation ability of HUVECs (Fig. 2I). These results suggested that silencing of circ_0001715 inhibited proliferation, but induced apoptosis in LUAD cell lines.

### Circ_0001715 acted as a sponge of miR-1322 in LUAD cell lines

We derived that circ_0001715 and miR-1322 had target binding sites by bioinformatics prediction software (Fig. 3A). Then we examined the expression of miR-1322 in LUAD, and the results showed that the expression of miR-1322 was significantly reduced in LUAD tissues and its expression had a negative correlation with circ_0001715 (Fig. 3B C). Lower levels of miR-1322 were obtained in LUAD cell lines (Fig. 3D). We further examined the interaction between circ_0001715 and miR-1322. First, dual luciferase reporter assay showed that transfection of miR-1322 mimics significantly reduced the luciferase activity of the circ_0001715 wild-type group, but not the circ_0001715 mutant-type group (Fig. 3E F). RNA pull-down assay results further indicated an interaction between circ_0001715 and miR-1322 in A549 and H1299 cells (Fig. 3G H).

**Fig. 7:**
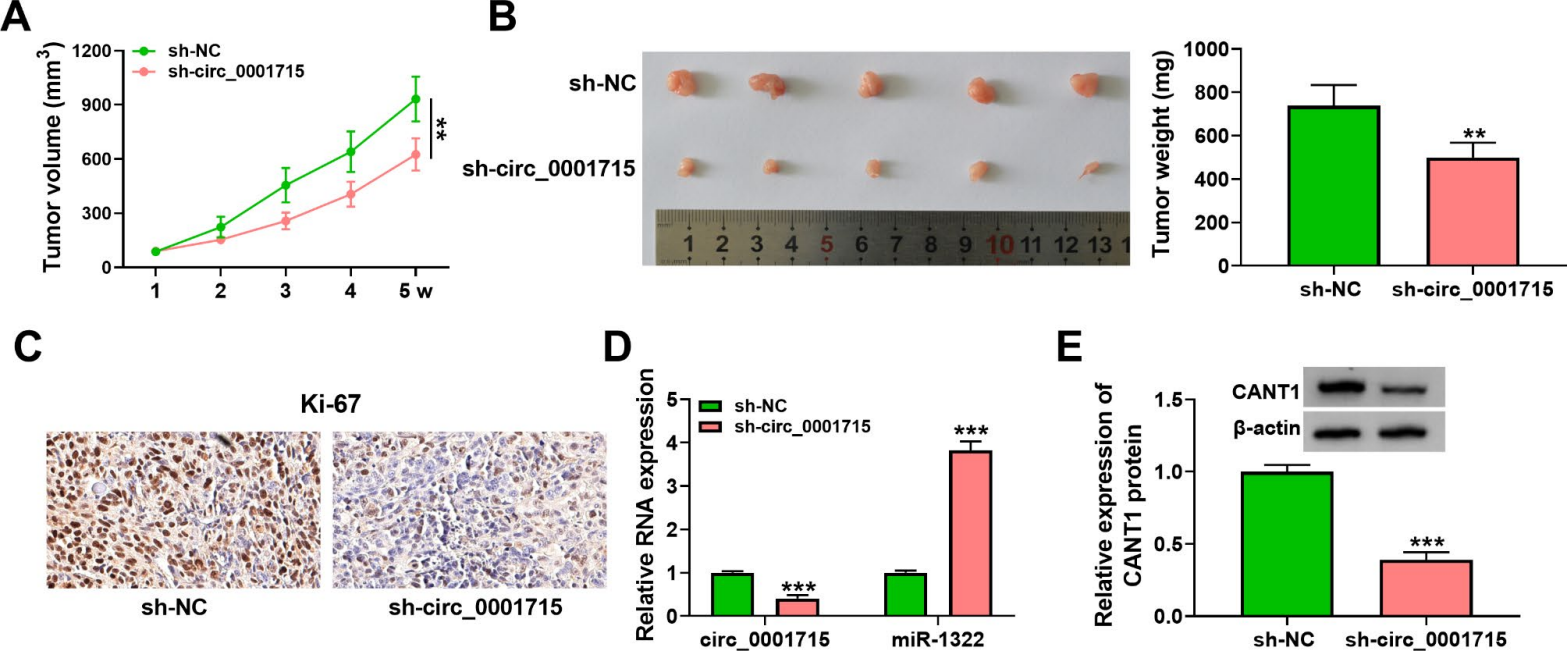
**Circ_0001715 silence hindered tumor growth** in vivo. Tumor volumes were measured every week (A). The presentative image of xenograft tumors was shown and tumor weights were measured (B). The expression of Ki-67 was evaluated by IHC (C). Relative miR-1322 expression and circ_0001715 expression in xenograft tumor tissues were measured by RT-qPCR (D). The expression of CANT1 in xenograft tumor tissues was assessed using western blot assay (E). **p < 0.01, ***p < 0.001

### Circ_0001715 promoted proliferation and inhibited apoptosis of LUAD cells via miR-1322

To verify the regulatory role of circ_0001715 and miR-1322 on LUAD cells function, we conducted a rescue assay. RT-qPCR results showed that the expression level of miR-1322 was reduced after transfection with miR-1322 inhibitor in A549 and H1299 cells (Fig. 4A). Clone formation assay results indicated that the inhibitory effect of silencing of circ_0001715 on cells colony forming ability was reversed by miR-1322 inhibitor in A549 and H1299 cells (Fig. 4B). The EDU assay and wound healing assay were also performed, and the results showed that low expression of circ_0001715 inhibited the proliferative effect of cells, but this effect was partially restored by the addition of miR-1322 inhibitor (Fig. 4C and D). And then, the reduced cell sphere formation efficiency and elevated cell apoptosis that was inhibited by si-circ_0001715 in LUAD cells were partly reversed by downregulation of miR-1322 in LUAD cells (Fig. 4E F). Finally, western bot assay was performed, and the changes in PCNA and Bax protein expression in A549 and H1299 cells induced by silencing of circ_0001715 were also reversed by miR-1322 inhibitor (Fig. 4G H). The lowered tube formation ability of HUVECs caused by circ_0001715 silencing was weakened after miR-1322 knockdown (Fig. 4I). In conclusion, circ_0001715 regulates the progression of LUAD cells through sponging miR-1322.

### MiR-1322 directly bound CANT1 to regulate the level of CANT1 in LUAD cell lines

Further, we performed predictive analysis by TargetScan database and GEPIA database and concluded that CANT1 was a downstream target of miR-1322 (Fig. 5A). RT-qPCR and western bot assay showed that CANT1 was significantly overexpressed in LUAD tissues (Fig. 5B). Moreover, CANT1 expression was negatively correlated with miR-1322 and positively correlated with circ_0001715 in LUAD tissues (Fig. 5C and D). CANT1 protein levels were elevated in LUAD tissues and cell lines (Fig. 5E F). Dual-luciferase reporter assay showed that miR-1322 mimic significantly decreased the luciferase activity of CANT1-3’UTR^WT^ (Fig. 5G H). The protein expression level of CANT1 was significantly increased after transfection of miR-1322 inhibitor in A549 and H1299 cells (Fig. 5I J). Furthermore, silencing of circ_0001715 was followed by a decrease in the protein expression level of CANT1, but the addition of miR-1322 inhibitor reversed the protein expression level of CANT1 (Fig. 5K L). Therefore, circ_0001715 could regulate CANT1 expression through miR-1322.

### Overexpression of CANT1 reversed the effect of circ_0001715 silence on the biological properties of LUAD cell lines

To further investigate whether circ_0001715 regulates the progression of LUAD cell lines through miR-1322/CANT1, we performed a rescue assay. Western bot assay showed a significant increase in CANT1 expression level in the pcDNA3.0-CANT1 group (Fig. 6A). Further clone formation assay, EDU assay and wound healing assay were performed and the results showed that silencing of circ_0001715 suppressed cell proliferation, whereas this effect could be reversed by high expression of CANT1 (Fig. 6B and D). Sphere formation assay showed that the cell sphere-forming efficiency inhibited by low expression of circ_0001715 was also reversed by overexpression of CANT1 in A549 and H1299 cells (Fig. 6E). In addition, we performed a flow cytometry assay, which showed that overexpression of CANT1 blocked apoptosis induced by knockdown of circ_0001715 (Fig. 6F). Finally, western bot assay verified that silencing of circ_0001715 suppressed PCNA protein expression, but promoted Bax protein expression in A549 and H1299 cells, and these effects could be reversed by overexpression of CANT1 (Fig. 6G H). Also, the upregulation of CANT1 impaired the decreased tube formation ability of HUVECs mediated by circ_0001715 silencing (Fig. 6I). Taken together, circ_0001715 could regulate the developmental process of LUAD through the miR-1322/CANT1 axis.

### Silencing of circ_0001715 inhibited LUAD tumor growth ***in vivo***

Finally, to validate the results of circ_0001715 in vitro assays, we performed in vivo experiments. First, we constructed a nude mouse tumor-forming model, and A549 cells stably expressing sh-NC or sh-circ_0001715 were injected subcutaneously into nude mice. The results showed that the tumor volume and tumor mass were significantly reduced in the sh-circ_0001715 group compared to the sh-NC group (Fig. 7A and B). Then IHC assay was performed, which showed that the expression of Ki-67, a proliferation marker, was also significantly reduced in the sh-circ_0001715 group (Fig. 7C). Finally, the expression of circ_0001715, circ_0001715, miR-1322 and CANT1 were measured after silencing of circ_0001715, and the results showed that the expression of circ_0001715 and CANT1 were significantly down-regulated, whereas the expression of miR-1322 was increased in sh-circ_0001715 group (Fig. 7D and E). In short, silencing of circ_0001715 could inhibit tumor growth in vivo.

## Discussion

In recent years, there are still great difficulties in the treatment of LUAD. Therefore, it is particularly important to explore the pathogenesis of LUAD [26, 27]. In our study, we found that circ_0001715 promoted LUAD development through the miR-1322 /CANT1 axis. Our functional results showed that circ_0001715 could act as a sponge for miR-1322 and then promote the expression of CANT1, thereby promoting the progression of LUAD.

In the introduction, we introduced the stable expression of circRNAs in cells. In addition, circRNAs are expressed in a variety of diseases, and abnormal expression is also evident in cancer, such as gallbladder cancer [28], bladder cancer [29], oral squamous cell carcinoma [30], hepatocellular carcinoma [31], colon cancer [32] and so on. Guo et al. found that circ_0001715 was significantly highly expressed in LUAD [15]. Interestingly, in our study on the function of circ_0001715 in LUAD cell lines, it was found that highly expressed circ_0001715 promoted cell progression, tube formation and cell proliferation was reduced and apoptosis rate was increased after silencing circ_0001715. We got the same results in vivo.

We further explored the action mechanism of circ_0001715 in LUAD. It has been reported in many studies that circRNAs could act as sponges of miRNAs and be involved in the development of cancer [18, 19, 29]. Therefore, we used bioinformatics software to perform a predictive analysis of the downstream miRNAs of circ_0001715 and concluded that the sequence of circ_0001715 contained binding sites that can potentially bind to miR-1322. As determined by luciferase activity assay and RNA pull-down assay, circ_0001715 and miR-1322 had an interactive relationship in cells. Hence, we identified that circ_0001715 can function further as a sponge for miR-1322.

It has been reported that miR-1322 was lowly expressed in LUAD [19], and the same result was obtained in our results. In our study, it was found that the silencing of circ_0001715 could inhibit cell proliferation and tube formation, as well as promote apoptosis. And the changes in cell biological characteristics caused by the downregulation of circ_0001715 could be partially reversed when miR-1322 inhibitors were co-transfected into the cells. In addition, we also found that circ_0001715 can regulate the expression of CANT1 through miR-1322. In addition, overexpression of CANT1 in cells could partially rescue the effects of circ_0001715 silencing on cell proliferation and cell apoptosis. The limitation of this paper is that it has not been explored whether circ_0001715 can play a role in LUAD by translating peptides as translation templates, which needs to be validated in the future.

In summary, this study suggested that circ_0001715 could sponge miR-1322 and up-regulate CANT1 expression, thereby accelerating the progression of LUAD. Therefore, circ_0001715 could be used as an effective biomarker and play a very important role in the treatment of LUAD.

### Electronic supplementary material

Below is the link to the electronic supplementary material.

Supplementary Material 1: Fig. 1. The mRNA levels of circ_0001715 and linear LIMK1 in A549 and H1299 cells treated with RNase R. ***p < 0.001.

## Data Availability

The analyzed data sets generated during the present study are available from the corresponding author on reasonable request.
