# Supplementary figures and images for: Circ_0001715 accelerated lung adenocarcinoma process by the miR-1322/CANT1 axis
Source: Diagn Pathol. 2023 Aug 8;18:91. doi: 10.1186/s13000-023-01348-2 (PMC10408075; doi:10.1186/s13000-023-01348-2)

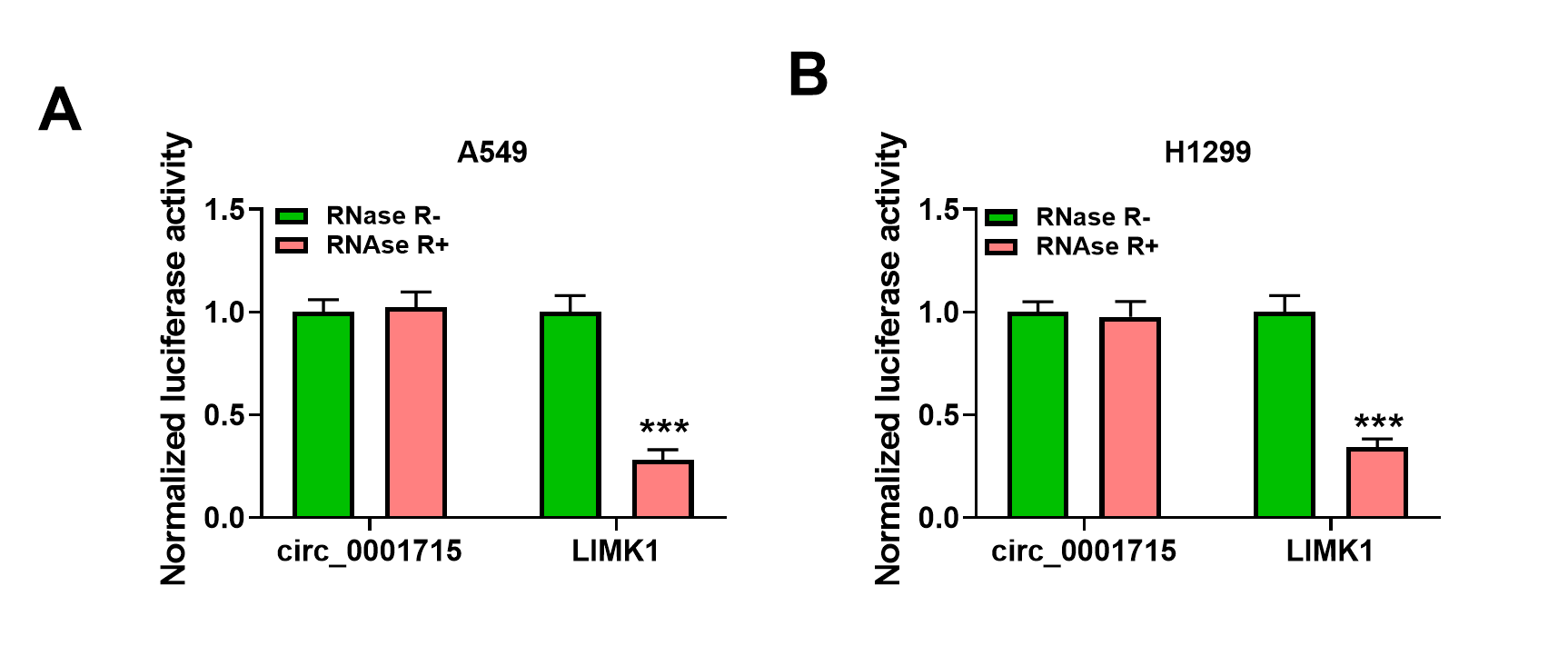

Supplement: Supplementary file 1 — Supplementary Material 1: Fig. 1. The mRNA levels of circ_0001715 and linear LIMK1 in A549 and H1299 cells treated with RNase R. ***p < 0.001. [file 13000_2023_1348_MOESM1_ESM.tif]
